# Trends of the prevalence rate of central lymph node metastasis and multifocality in patients with low-risk papillary thyroid carcinoma after delayed thyroid surgery

**DOI:** 10.3389/fendo.2024.1349272

**Published:** 2024-04-04

**Authors:** Pei Wang, Zhizhong Dong, Shuyan Zhao, Yanjun Su, Jianming Zhang, Yunhai Ma, Chang Diao, Jun Qian, Ruochuan Cheng, Wen Liu

**Affiliations:** ^1^ Department of Radiation Oncology, Cancer Institute, The First Affiliated Hospital, and College of Clinical Medicine of Henan University of Science and Technology, Luoyang, China; ^2^ Department of Medical Imaging, The First Affiliated Hospital of Kunming Medical University, Kunming, China; ^3^ Department of Thyroid Surgery, Clinical Research Center for Thyroid Disease of Yunnan Province, The First Affiliated Hospital of Kunming Medical University, Kunming, China

**Keywords:** papillary thyroid carcinoma, low-risk thyroid cancer, active surveillance, lymph node metastasis, multifocality

## Abstract

**Background:**

Active surveillance has been an option for patients with low-risk papillary thyroid carcinoma (PTC). However, whether delayed surgery leads to an increased risk of local tumor metastasis remain unclear. We sought to investigate the impact of observation time on central lymph node metastasis (CLNM) and multifocal disease in patients with low-risk PTC.

**Methods:**

Patients who were diagnosed with asymptomatic low-risk PTC, and with a pathological maximum tumor size ≤1.5 cm by were included. The patients were classified into observation group and immediate surgery group, and subgroup analyses were conducted by observation time period. The prevalence of CLNM, lymph node (LN) involved >5, multifocal PTC and bilateral multifocal PTC were considered as outcome variables. The changing trend and risk ratio of prevalence over observation time were evaluated by Mann-Kendall trend test and Logistics regression.

**Results:**

Overall, 3,427 and 1,860 patients were classified to the observation group and immediate surgery group, respectively. Trend tests showed that decreasing trends both on the prevalence of CLNM and LN involved >5 over the observation time, but the difference was not statistically significant, and the prevalence of multifocal PTC and bilateral multifocal PTC showed the significant decreasing trends. After adjustment, multivariate analysis showed no statistically significant difference between observed and immediate surgery groups in the four outcome variables.

**Conclusion:**

In patients with subclinical asymptomatic low-risk PTC, observation did not result in an increased incidence of local metastatic disease, nor did the increased surgery extent in patients with delayed surgery compared to immediate surgery. These findings can strengthen the confidence in the active surveillance management for both doctors and patients.

## 1 Introduction

Thyroid cancer is one of the fastest-growing solid tumors worldwide, with more than a third of the cases occurring in China, where over 730,000 new diagnoses were made in the past five years (1). Papillary thyroid carcinoma (PTC) accounts for more than 80% of thyroid cancers and is the histological type with the best prognosis (2, 3). The widespread use of physical examinations and ultrasound is the main reason for the explosive increase in thyroid cancer incidence rate, and it has led to the detection of some small subclinical PTCs that may not affect the patient’s life quality or survival. Overdiagnosis and overtreatment of PTC have raised concerns not only in the clinical medical field, but also in the society at large (4, 5).

Active surveillance (AS) is a management strategy for subclinical tumors that can reduce the risks of unnecessary treatment side effects and surgical complications (6, 7). Many clinical studies have been conducted in recent years to compare AS with immediate surgery as management options for low-risk PTC. Some cohorts have changed the threshold for AS management to include PTC < 1.5 cm, as safety and excellent prognosis are confirmed by long-term follow-up data (8). More and more clinical practice guidelines in developed countries with high medical standards, such as the United States, Europe, Japan, and South Korea, recommend AS as the first-line management strategy for low-risk PTC (8).

Two criteria for surgery in the AS management of low-risk PTC are tumor progression and lymph node metastasis detected by ultrasound (9). Ultrasound measurement can effectively differentiate between “resting cancer” and “progressive cancer” based on the tumor size over time during AS. Central lymph node metastasis (LNM) is a common clinical feature of PTC. According to the largest AS cohort from Kuma Hospital, only 1.5% of 1295 low-risk papillary thyroid microcarcinoma (PTMC) patients developed new LNM during an average follow-up of 60 months (10). In contrast, another study by the same author from the same institution during the same period reported a postoperative pathological LNM rate of 50.5% among 594 surgical patients with similar clinical characteristics to the AS cohort (11). The sensitivity of ultrasound detection of central LNM is only 16.8%-38.0% due to thyroid obstruction (12–14). The paradox between low-risk thyroid cancer observation and high LNM prevalence is a major concern for patients and a source of doubt for some scholars regarding the AS plan (15–17). Moreover, some patients may change their monitoring intentions due to concerns about new cancer foci during observation. The origin of multifocal PTC remains controversial, as it can be explained by two different mechanisms: either multiple cancer foci have different clonal origins, or a single clonal origin cancer focus spreads intraglandularly (18). An increase in the incidence of multifocal cancer during observation may imply that monitoring may lead to more extensive surgery and worse patient prognosis if surgery is delayed, regardless of the origin. Therefore, a necessary prerequisite for conducting and promoting AS plans may be to evaluate whether observation is associated with increased LNM and multifocal cancer. Existing AS cohort studies have very few outcome events (surgery) and thus insufficient sample sizes to evaluate LNM and multifocal disease status in pathological examinations. Retrospective large-sample cohorts may be the best research method to evaluate the clinical impact of observation time on these two aspects under existing conditions.

This study aims to evaluate the following aspects in subclinical low-risk PTC detected by physical examination: (1) the correlation between observation time and the incidence of central LNM and LNM>5 in postoperative pathological examinations, which indicate the risk of new central micro-metastasis and existing m-LNM progression during AS; (2) the correlation between observation time and the incidence of multifocal cancer and bilateral multifocal cancer in postoperative pathological examinations, which reflect the risk of worsened prognosis and increased surgical extent during AS.

## 2 Materials and methods

### 2.1 Data sources and study design

This study used the thyroid cancer database of the First Affiliated Hospital of Kunming Medical University, which contained all thyroid cancer surgical cases from January 2007 to December 2020. The main symptoms and duration of the patient’s visit at admission, as well as the disease’s occurrence, evolution, and treatment, are recorded in detail in the electronic medical record. Since benign nodules and PTC have different genetic backgrounds and differentiation origins, thyroid nodules (benign or malignant) do not change their nature with observation time (19, 20). Pathologically confirmed PTC usually has a determined malignant nature, even if the imaging malignant signs are unclear at the initial discovery. Thus, a retrospective review of the clinical data of PTC patients can evaluate the impact of nodule observation time on LNM and multifocality.

This study included all low-risk PTC patients in the database who were potentially suitable for AS management between January 2007 and December 2020. The inclusion criteria were: (1) asymptomatic thyroid cancer detected by health check-ups; (2) age ≥ 18 years; (3) thyroidectomy and prophylactic central lymph node dissection (CLND); (4) no clinical or imaging evidence of LNM before surgery; (5) postoperative paraffin pathology confirmed PTC with a maximum tumor diameter ≤ 1.5cm. The exclusion criteria were: (1) coexistence with other types of thyroid cancer; (2) prior history of head and neck surgery or radiation; (3) postoperative pathological examination revealed exthyroid infiltration; (4) distant metastasis. Cases where the tumor location was close to but not infiltrating the capsule under ultrasound were not excluded, as they may not affect the evaluation of lymph nodes. The range of CLND is defined by the following margins (21): The boundaries of the level VI lymph nodes are defined superiorly by the hyoid bone, inferiorly by the sternal notch, laterally by the medial aspect of the carotid sheath, posteriorly by the prevertebral fascia, and anteriorly by the superficial layer of the deep cervical fascia. The unilateral central lymph node dissection involves the lesion’s side and lymph nodes in front of the larynx and trachea. All reports are jointly issued by two experienced pathologists. The main aim of this study was to evaluate the safety of AS regarding LNM and intraglandular metastasis-related characteristics in low-risk PTMC management. Considering that PTC with a diameter of 1cm may increase in tumor size during AS, and that AS mainly relies on ultrasound to measure the tumor diameter line, but the tissue shrinks after postoperative paraffin fixation, the tumor size may be underestimated. Therefore, we set the tumor size threshold for inclusion cases at 1.5 cm.

This study received ethical approval from the Ethics Committee of the First Affiliated Hospital of Kunming Medical University (Ethics Approval No. 2017-17), and all patients provided informed consent.

### 2.2 Evaluation of observation time

When the patient is admitted, the resident physician will identify the target cancer lesion for the planned surgical treatment based on the outpatient medical record. The patient will also be asked about the duration since the thyroid nodule was detected, and it will be recorded in the admission medical record precisely by month or year. For example, records such as: thyroid nodule detected for more than 1 month, 6 months, more than 1 year, etc. Two thyroid specialists will jointly record and cross-verify the patient’s epidemiological and clinical pathological characteristics, including observation time.

The study cohort was split into two groups: the observation group and the non-observation group, to examine the effect of observation time on the incidence of LNM and multifocal cancer. The observation group consisted of asymptomatic thyroid nodules detected in physical examinations, but not operated on within 2 months. This study performed additional analyses to investigate the association between different observation times and the incidence of LNM and multifocal cancer in low-risk PTC. Observation time was categorized into seven subgroups: non-observation group (immediate surgery); observation for 2-6 months, 6-12 months, 12-24 months, 24-36 months, 36-60 months, and ≥ 60 months. LNM and multifocal cancer were evaluated based on the pathological report, with multifocal cancer defined as having two or more cancer foci.

### 2.3 Statistical analysis

We performed statistical analysis using R version 3.6.3. We described normally distributed measurement data using mean ± standard deviation, skewed distribution using median (IQR), and count data using rate and composition ratio. We evaluated the trend of outcome indicators using the Mann-Kendall trend test, where *Z* < 0 indicated a decrease, *Z* > 0 indicated an increase, and *P* < 0.01 indicated a significant trend change. We tested hypotheses of count data using the χ2 test, and of normally distributed measurement data using the independent sample t-test. We performed multifactor analysis using logistic regression, where *P* < 0.05 indicated statistically significant differences.

## 3 Results

### 3.1 Cohort characteristics

**Figure 1:**
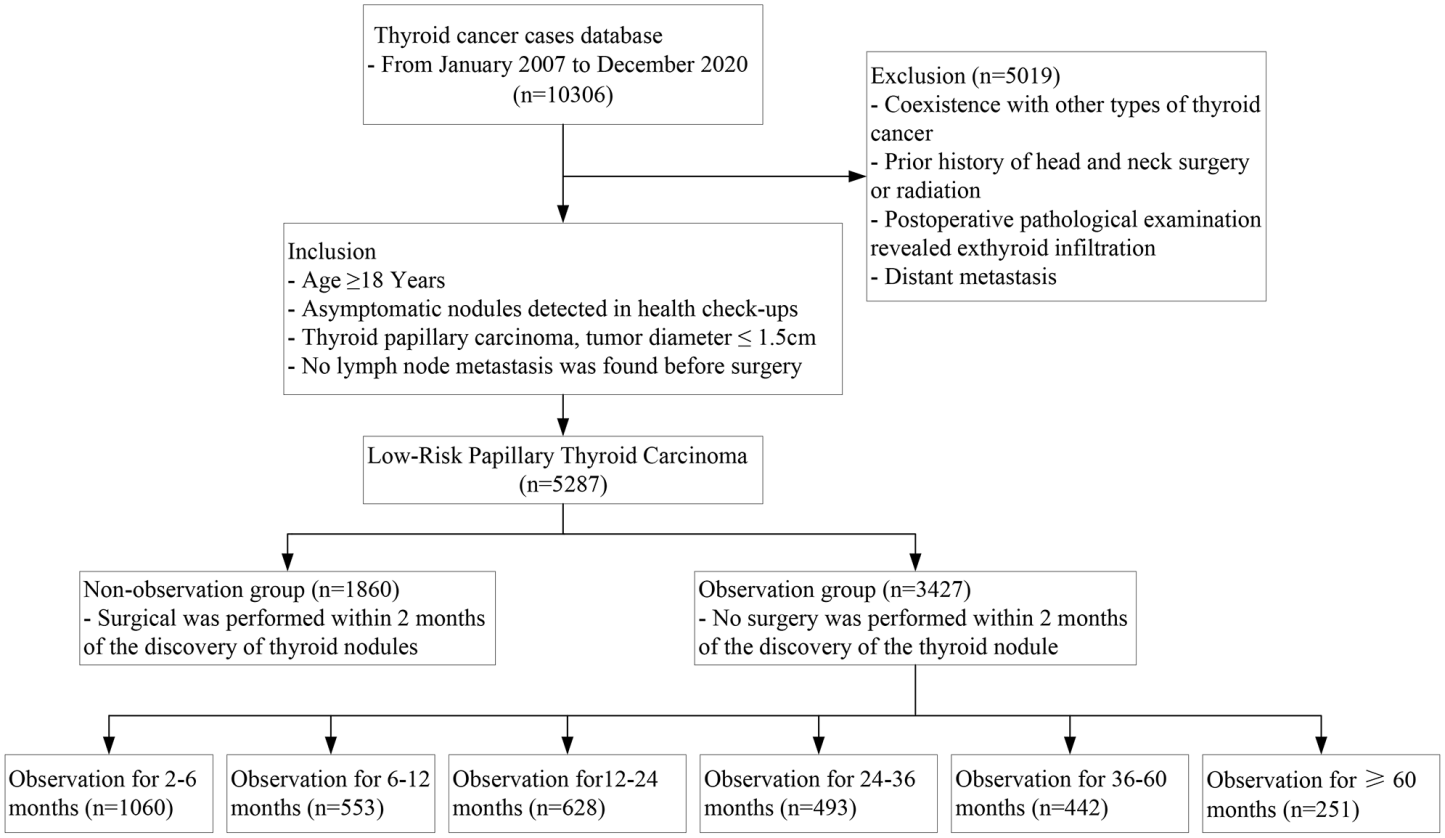
Flow chart of the included patients.

Out of the 5287 low-risk PTCs that met the inclusion criteria, 1860 cases (35.2%) had immediate surgery (within 2 months of nodule detection); 3427 cases (64.8%) had surgery after nodule observation, with a median observation time of 12 (4–24) months, and more than 2 years of observation time in 34.6% of the observation group cases. (**Figure 1**) Postoperative pathological examinations revealed central LNM in 1582 cases (29.9%), with central LNM>5 in only 2.5% of cases; multifocal PTC in 31.1% of cases and bilateral multifocal PTC in 20.5% of cases. **Table 1** shows the baseline characteristics of the cohort, which had significant differences in the incidence of central LNM and multifocal cancer between the observation group and the non-observation group.

**Table 1:** Characteristics of the study population.

Characteristic	All Patients (N=5287)	Observation group(N=3427)	Non- observation group(N=1860)	P value
**Age (years), median (IQR)**	43.0 (36.0-51.0)	44.0 (36.0-51.0)	43.0 (36.0-50.0)	0.079
				0.583
< 40 years	1931 (36.5%)	1250 (36.5%)	681 (36.6%)	
40 – 59 years	2907 (55.0%)	1876 (54.7%)	1031 (55.4%)	
≥ 60 years	449 (8.5%)	301 (8.8%)	148 (8.0%)	
**Sex, Female**	4184 (79.1%)	2721 (79.4%)	1463 (78.7%)	0.525
**Tumor size (cm), median (IQR)**	0.6 (0.4-1.0)	0.60 (0.4-1.0)	0.60 (0.5-1.0)	0.002
				0.017
≤ 0.5	2237 (42.3%)	1499 (43.8%)	738 (39.7%)	
0.6-1.0	2239 (42.4%)	1413 (41.2%)	826 (44.4%)	
> 1.0 – 1.5	811 (15.3%)	515 (15.0%)	296 (15.9%)	
**Multifocality**	1643 (31.1%)	1032 (30.1%)	611 (32.8%)	0.040
**Bilateral multifocality**	1085 (20.5%)	682 (19.9%)	403 (21.6%)	0.129
**Hashimoto’s thyroiditis**	1404 (26.6%)	915 (26.7%)	489 (26.3%)	0.748
**Surgery**				0.004
Lobectomy	2222 (42.0%)	1490 (43.5%)	732 (39.4%)	
Bilateral thyroidectomy ^†^	3065 (58.0%)	1937 (56.5%)	1128 (60.6%)	
**CLND**				<0.001
Complete CLND	2934 (55.5%)	1833 (53.5%)	1101 (59.2%)	
Ipsilateral CLND	2353 (44.5%)	1594 (46.5%)	759 (40.8%)	
**LNs examined, median (IQR)**	7 (4-11)	7 (4-10)	7 (4-11)	0.055
**Positive LNs, median (IQR)**	2(1-3)	2(1-3)	2(1-3)	0.749
**CLNM**	1582 (29.9%)	984 (28.7%)	598 (32.2%)	0.009
**CLNM > 5 Nodes**	130 (2.5%)	88 (2.6%)	42 (2.3%)	0.482
**Duration of observation (months), median (IQR)**	NA	12.0(4.0,24.0)	NA	

^†^The bilateral thyroidectomy group includes total/near-total thyroidectomy (2886 cases), subtotal thyroidectomy (2 cases), and unilateral lobectomy + isthmusectomy + partial lobectomy of the contralateral lobe (177 cases).

IQR, interquartile range; CLND, central lymph node dissection; LN, Lymph node; CLNM, central lymph node metastasis.

### 3.2 Observation time and lymph node metastasis

**Figure 2:**
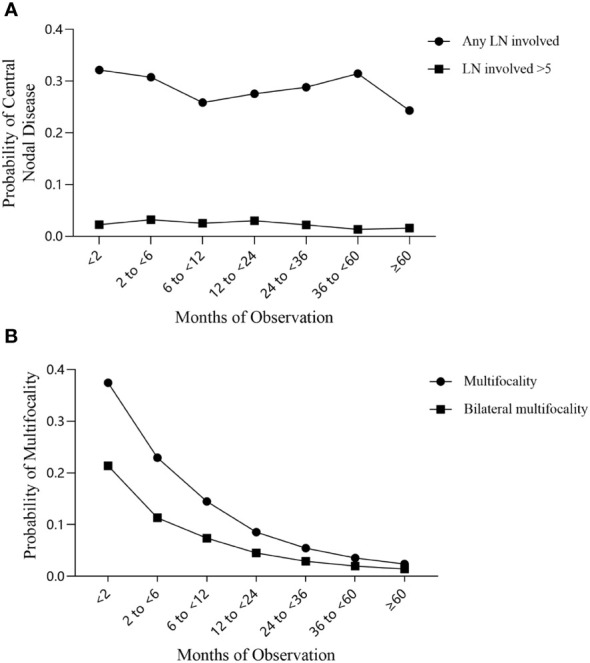
Trends in the incidence of central lymph node metastasis and multifocal PTC over the observation period. **(A)** Central lymph node metastasis; **(B)** Multifocal PTC.

We evaluated the trend of central lymph node status in the 3427 observation group cases and 1860 non-observation group cases from the study cohort. The trend test results indicated that the incidence of both central LNM and LNM>5 decreased with longer observation time (*Z*= -1.608 and *Z*= -0.901), but the differences were not statistically significant (*P*= 0.108 and *P*= 0.368) (**Figure 2A**).

**Figure 3:**
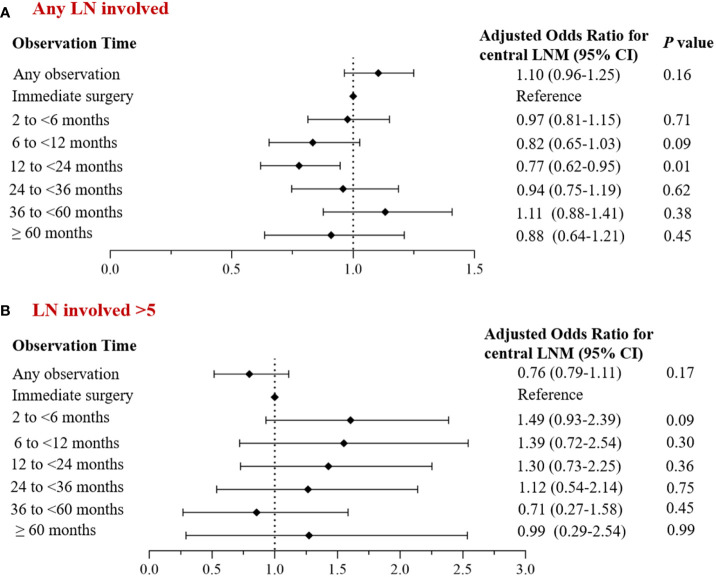
Multivariate analysis of the corrected incidence of central LNM and observation time. **(A)** Incidence of central lymph node metastasis; **(B)** Incidence of central lymph node metastases >5.

Univariate analysis revealed group differences in tumor size, multifocal cancer, and the extent of central lymph node dissection between cases with and without observation. After adjusting for baseline demographic data (age, gender) and clinical characteristic factors, we found no statistically significant difference in the incidence of central LNM between the overall observation group and the non-observation group (adjusted OR: 1.10; 95% *CI*: 0.96~ 1.25; *P*= 0.16), although it slightly increased in the former group. Only the observation time subgroup of 12-24 months had a significant decrease in the incidence of central LNM (adjusted OR: 0.77; 95% *CI*: 0.62~ 0.95; *P*= 0.01). The incidence of central LNM>5 also decreased in the overall observation group, but not significantly (adjusted OR: 0.76; 95% *CI*: 0.79~ 1.11; *P*= 0.17) (**Figure 3**); none of the observation time subgroups had a significant change in the incidence of central LNM>5.

### 3.3 Observation time and multifocality

We used the trend test to evaluate disease multifocality and found that the incidence of both multifocal PTC and bilateral multifocal PTC decreased significantly with longer observation time (both *Z*= -3.004; *P*= 0.003) (**Figure 2B**).

**Figure 4:**
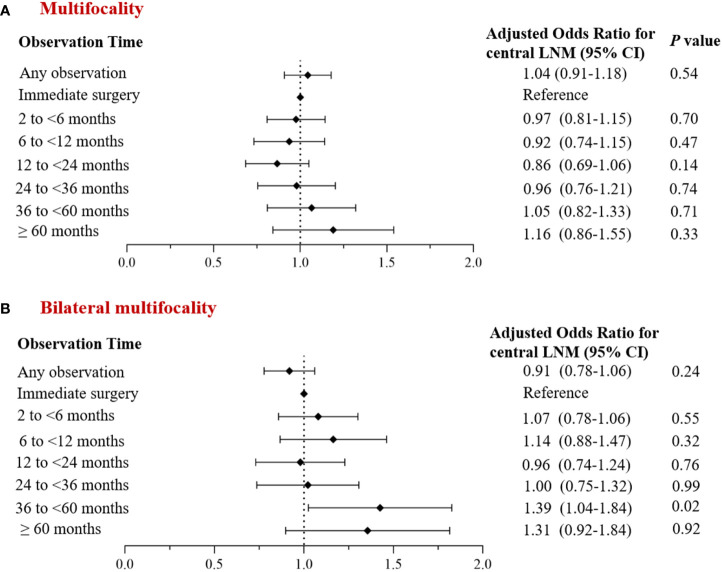
Multivariate analysis of the corrected incidence of multifocal PTC and observation time. **(A)** Incidence of multifocal cancer; **(B)** Incidence of bilateral multifocal cancer.

Univariate analysis revealed group differences in tumor size and thyroidectomy extent between cases with and without observation. After adjusting for baseline demographic data (age, gender) and clinical characteristic factors, we found no significant difference in the incidence of multifocal PTC and bilateral multifocal PTC between the overall observation group and the non-observation group (adjusted OR: 1.04; 95% *CI*: 0.91~ 1.18; *P*= 0.54 and adjusted OR: 0.91; 95% *CI*: 0.78~ 1.06; *P*= 0.24), although it decreased in the former group (**Figure 4**).

## 4 Discussion

Early screening, diagnosis, and treatment are essential for tumor management, as delayed diagnosis and treatment may worsen tumor stage and long-term prognosis. However, given the limited accuracy of non-invasive examinations in distinguishing benign nodules from PTC, research on the relationship between observation time and outcomes in PTC remains constrained. Recent breakthroughs in both clinical and basic research have provided opportunities to investigate this issue. A multicenter prospective clinical observation study involving 992 patients with benign thyroid nodules diagnosed through ultrasound and cytology found that only 5 original nodules were diagnosed as thyroid cancer after 5 years of follow-up (20). Additionally, another study utilizing whole-exome sequencing and/or transcriptome sequencing data confirmed the unique molecular characteristics and genetic backgrounds of benign nodules, distinct from PTC (19). PTC usually has a benign course and a favorable prognosis, but the effects of the interval from tumor detection to surgical treatment (observation) on long-term outcomes are disputed. Jeon et al.’s (22) study found no group differences in structural disease recurrence after matching for clinical characteristics by delaying surgery ≤ 6, 6-12, and > 12 months, supporting the use of AS management for PTMC. A study from NCDB reported that delaying surgery > 180 days increased mortality (aHR:1.94, 95%CI:1.68-2.24) (23). However, evidence for the association between observation time and invasive pathological features is still lacking.

Lymphatic metastasis is a common mode of dissemination for epithelial malignant tumors. Tumor cells invade the lymphatic vessels and drain to the local lymph nodes with the lymph fluid, forming metastatic foci. A questionnaire survey of AS patients from Japan reported that “tumor metastasis to other sites” was their main concern (16). Many reviews from China also expressed worries about LNM during AS (24–26). According to the 8th edition staging system of the American Joint Committee on Cancer (AJCC), LNM elevates the disease stage of PTC patients ≥ 55 years old from stage I to stage III (27). Moreover, the American Thyroid Association Thyroid Cancer Lymph Node Surgery Working Group stated that compared with preoperative clinically evident (cN1 stage) local regional lymph node metastasis, small-volume subclinical m-LNM (pN1 stage) has a very low recurrence risk; but as the number of metastases increases, so does the median recurrence risk significantly [< 5 nodes 4% (range: 3%-8%) vs. > 5 nodes 19% (range: 7%-21%)] (28). LNM is not only an independent risk factor for reduced survival rate in elderly patients (29, 30), but also associated with an additional increase in mortality risk in PTC patients aged < 45 years old with LNM> 5, according to a comprehensive analysis based on NCDB and SEER data (31). Therefore, the incidence of LNM>5 is an indicator for assessing recurrence and death risk. As the observation period extends, patients inevitably age, so new m-LNM or micro-metastasis progression to the next station (increased number of lymph node metastases) may imply delayed treatment for those low-risk PTC patients. This study, based on a large sample retrospective cohort, revealed for the first time that in asymptomatic low-risk PTC, observation did not increase LNM incidence and LNM>5 incidence in all patients and ≥ 55-year-old patients. These findings suggest that: (1) In asymptomatic low-risk PTC, the presence of clinically occult (cN0) lymph node micro-metastasis is determined at the onset of tumor development, and new primary tumor metastasis to local lymph nodes may not occur during the observation process. Thus, the indolent behavior of PTC may be reflected not only in its tumor growth state but also in its tumor cell lymphatic propensity. (2) In asymptomatic PTC with lymph node micro-metastasis (pN1 stage), lymph node metastases may not continue to spread to regional or distant lymph nodes. Lymph node micro-metastases have similar indolent biological features as primary tumors and may lack the ability to persistently disseminate within the lymphatic system.

Multifocality in thyroid cancer means having two or more cancer lesions in the thyroid gland at the same time, often as <1cm microcarcinomas. Multifocality occur in 18%-87% of PTCs, varying by different epidemiological characteristics and detection methods (32, 33). A recent meta-analysis of 26 studies with 33,976 patients found that multifocal PTC patients had a significantly higher recurrence rate than unifocal PTC patients (pooled HR: 1.81; 95% *CI*: 1.52~ 2.14; *P*< 0.01), but similar disease-specific survival rates (pooled HR: 1.19; 95% *CI*: 0.85~ 1.68; *P*= 0.31) (34). Another study reported that within one year after surgery, 42% of recurrences happened, and most of them (71 out of 92 cases) were tumor residues rather than “true” recurrences (35).

Multifocal PTC suggests a high level of genetic heterogeneity, and intrathyroidal spread may reflect the metastatic ability of the main tumor, leading to more intensive treatment. Current clinical practice guidelines often advise that multifocal PTC, especially bilateral multifocal PTC, should have total thyroidectomy as the initial surgical treatment (36). Thus, changes in the incidence of multifocal PTC during the observation period may result in a larger surgical extent and a worse prognosis in delayed surgery for AS cases, which also implies that patients have to face a higher risk of surgical complications and lifelong endocrine therapy. After adjusting for factors such as age, gender, tumor size, and thyroidectomy extent, the analysis found no significant change in the incidence of multifocal and bilateral multifocal PTC with observation time. This implies that during AS, the surgical extent will not increase due to a higher multifocality risk in long-term delayed surgery. This implies that during the AS, the increased risk of multifocality will not lead to extended surgical scope in the long-term delayed surgery.

This study has some limitations: First, it is based on retrospective cohort data and may have selection bias. Prospective AS cohorts have very few surgical cases, making it impossible to fully assess the pathological status of lymph nodes. And prospective studies may not be feasible for evaluating the incidence of LNM. Moreover, although a significant number of cases undergoing prophylactic CLND were included in the study cohort, the primary focus of this research is not to advocate for or against prophylactic CLND. Instead, this study confirms the inert behavior of metastatic lymph nodes in certain cases of PTC. We advocate for individualized decisions regarding prophylactic CNLD based on both physician and patient characteristics. Second, thyroid nodules in some patients may have been present for a while before detection, creating confounding factors in the observation period of this study. However, national or regional universal neck ultrasound screening is not recommended, and this confounding bias may affect all studies on observation time, including prospective cohorts. Increasing the sample size may help reduce confounding bias. Third, retrospective epidemiological studies based on patient reports may have recall bias, which may impair the accuracy of observation time records. However, in this study, all thyroid nodules were detected in physical examinations and usually had paper and electronic reports. Therefore, we think that recall bias may not influence the recording of observation time much. Fourth, although many high-quality studies indicate that the diameter of metastatic lymph nodes is strongly associated with the prognosis of PTC (9, 29, 37), only a few patients had the maximum diameter of metastatic lymph nodes measured in pathological examinations at our center, and this study could not assess the effect of observation time on the severity of metastatic lymph nodes. Finally, the research data came from a single-center database and could not exclude selection bias, so other cohorts are needed to confirm the findings of this study.

## 5 Conclusion

To conclude, subclinical asymptomatic low-risk PTC may have central lymph node micro-metastasis at the disease onset, but observation will not increase the lymph node metastasis rate or the incidence of multifocal cancer and bilateral multifocal cancer. In cases with lymph node micro-metastasis, the metastatic foci may also behave indolently and will not spread further to regional or distant lymph nodes. These findings may reduce patients’ worries about tumor metastasis and dissemination, enhance doctors’ confidence in AS management, and facilitate the adoption of AS programs and the advancement of non-surgical treatments, which have some value for achieving precise diagnosis and treatment of low-risk PTC.

## Data availability statement

The original contributions presented in the study are included in the article/supplementary material. Further inquiries can be directed to the corresponding authors.

## Ethics statement

The study was approved by the Ethics Committee of the First Affiliated Hospital of Kunming Medical University. The patients/participants provided their written informed consent to participate in this study. Written informed consent was obtained from the individual(s) for the publication of any potentially identifiable images or data included in this article.

## Author contributions

PW: Writing – review & editing, Writing – original draft, Funding acquisition, Formal analysis, Data curation. ZD: Writing – review & editing, Writing – original draft, Investigation, Formal analysis, Data curation. SZ: Formal analysis, Validation, Writing – review & editing. YS: Writing – review & editing, Supervision, Formal analysis. JZ: Writing – review & editing, Visualization, Methodology. YM: Writing – review & editing, Visualization, Validation. CD: Writing – review & editing, Formal analysis, Data curation. JQ: Writing – review & editing, Methodology, Investigation. RC: Writing – review & editing, Writing – original draft, Supervision, Project administration, Data curation, Conceptualization. WL: Writing – review & editing, Writing – original draft, Supervision, Project administration, Funding acquisition, Data curation, Conceptualization.
